# Atherosclerosis Might Be Responsible for Branch Artery Disease: Evidence From White Matter Hyperintensity Burden in Acute Isolated Pontine Infarction

**DOI:** 10.3389/fneur.2018.00840

**Published:** 2018-10-09

**Authors:** Lixin Zhou, Ming Yao, Bin Peng, Yicheng Zhu, Jun Ni, Liying Cui

**Affiliations:** ^1^Department of Neurology, Peking Union Medical College Hospital, Peking Union Medical College and Chinese Academy of Medical Sciences, Beijing, China; ^2^Neuroscience Center, Chinese Academy of Medical Sciences, Beijing, China

**Keywords:** isolated pontine infarction, etiological classification, branch atherosclerotic disease, white matter hyperintensity, SMART study

## Abstract

**Objective:** To investigate an MRI-based etiological classification for acute isolated pontine infarcts and to assess differences in vascular risk factors, clinical characteristics and WMH burden among the etiological subtypes.

**Methods:** All participants from SMART cohort with DWI-proven acute isolated pontine infarcts (AIPI) were included and categorized into 3 groups: large-artery-occlusive disease (LAOD), basilar artery branch disease (BAD), and small vessel disease (SVD), according to basilar artery atherosclerosis severity and lesion extent of the transverse axial plane. The vascular risk factors and 6-month functional outcome was analyzed among 3 groups.

**Results:** Of the 1129 patients enrolled, 175 had AIPI. BAD was the most frequent subtype of AIPI (46.3%), followed by SVD (36.0%) and LAOD (17.7%). Neurological impairment on admission was more severe in the LAOD group, followed by BAD. The BAD group had greater frequencies of female sex, hypertension, diabetes mellitus compared to the SVD group (*P* < 0.05). NIHSS on admission were significantly higher in the BAD group as compared with the SVD group (*P* < 0.001), but no difference was found between BAD and LAOD group. Poor outcome (mRS≥3) was found in only 13.7% of patients at 6-month post-stroke and there was no difference among 3 groups. WMH severity was significant higher in the SVD group compared to the BAD group for the deep subcortical region; however, there was no difference for the periventricualr region. There was no significant difference in either DWMH or PVWMH severity between the BAD and LAOD groups.

**Conclusion:** BAD is the most frequent etiology of AIPI followed by SVD and LAOD. WMH burden, vascular risk factors and clinical characteristics in BAD group were more similar to the LAOD group, rather than to the SVD group, suggesting the atherothrombotic nature of BAD.

Acute isolated pontine infarct (AIPI) is the most common subtype of posterior circulation infarct. AIPI can be caused by various stroke etiologies, including branch atheromatous disease (BAD), small vessel disease (SVD), and large artery occlusion disease (LAOD) (1). The seminal clinico-pathological studies by Fisher and Caplan first described branch atheromatous disease (BAD) and distinguished it from lacunar infarct caused by small vessel lipohyalinotic degeneration in patients with pontine infarct (2, 3). Since routine imaging techniques are unable to depict small vessel changes, BAD is mostly defined by indirect imaging features. Multiple contemporary series using magnetic resonance images (MRI) have examined the correlation between pontine infarct etiology and infarct morphology (4, 5). The morphologic characteristics were considered to differentiate BAD related infarct from small vessel disease (SVD). Pontine infarct was most often related to BAD when the lesion abuts on the basal surface (paramedian pontine infarct, PPI), whereas lacuna pontine infarct (LPI) presented with a small lesion not extent to the basal surface was usually attributed to SVD (5–7). However, there is no standard etiological classification based on infarct morphology nor has this classification system been examined in large cohorts. Further, whether the etiological subtypes of AIPI bears any clinical and other neuroimaging significance is unclear.

In fact, currently there is no consensus as to whether BAD should be included either in small vessel or in large vessel intracranial disease (8, 9), which further impact the clinical prevent decision. Some studies reported BAD as one of the stroke mechanisms caused by intracranial atherosclerotic disease and characterized by a milder degree of large artery stenosis (7, 10, 11). However, some studies demonstrated that small vessel abnormalities might be involved in BAD pathophysiology (12). White matter hyperintensities (WMH), a typical marker of SVD detected on MRI, is prevalent in acute stroke population. It is reasonable and valuable to investigate the difference in WMH burden between SVD and BAD to identify the underlying pathophysiology of BAD. Therefore, we hypothesized that pathophysiological mechanism of BAD may most attributed to atherosclerosis, which would be reflected by less WMH burden in the BAD group compared to the SVD groups and similar to LAOD.

Thus, the aim of the present study is to investigate the MRI-based etiological classification for AIPI and to assess differences in vascular risk factors, clinical characteristics, and WMH burden among the patients with different etiological subtypes.

## Methods

### Study population

Data were collected from SMART study, which was a multicenter, randomized controlled trial to evaluate the feasibility and efficacy of a guideline-based program in secondary stroke prevention across 47 hospitals in China. A detailed description of study protocol and main results of SMART has been published elsewhere (13, 14). A total of 3821 participants with a first-ever ischemic stroke within 30 days were enrolled in SMART study between April 2008 and December 2010. Of them, MRI-proven acute ischemic stroke was identified in 1129 participants from SMART study. Acute isolated pontine infarcts proved by DWI-MRI were identified (*n* = 189). Of them, the patients with probable etiology of cardioembolism (*n* = 4) and lack of complete images for review (*n* = 10) were further excluded, leaving 175 for this sub-group analysis.

Written informed consent was obtained from all participants. The study was approved by the central ethics committee of the principal study center at Peking Union Medical College Hospital and by the ethics committees at the participating study sites.

### Brain MRI analysis and etiological classification of AIPI

Pontine lesions were estimated by two board-certified neurologists (ZLX, ZMY) who were blinded to the clinical parameters. We reviewed axial sections of the DWI sequences to capture the infarct morphology. Basically, isolated pontine infarcts were divided into two groups based on whether the lesion morphology on MRI extent to the anterior surface of the pons or not: paramedian pontine infarct (PPI) and lacunar pontine infarcts (LPI) according to the previous published studies (5, 6). Inter-rater reliability for the classification of the infarct morphology was assessed using a subset of 60 random study subjects. Kappa value for the inter-rater agreements was 0.84.

**Figure 1:**
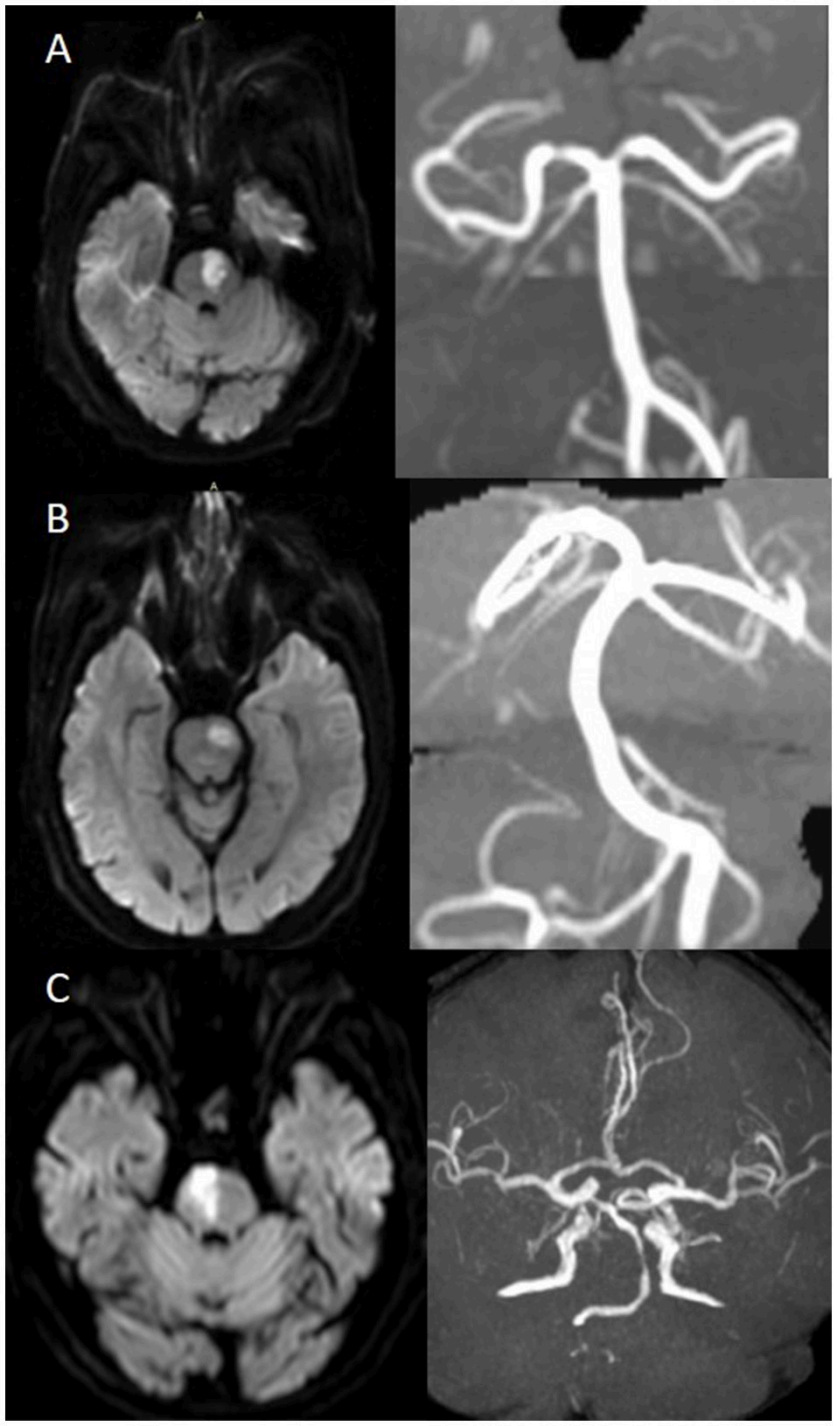
**(A)** BAD. DWI showing a substantial infarct that reached the pontine surface (PPI). Basilar artery stenosis was not observed on MRA. **(B)** SVD. DWI showing a lacunar pontine infarct (LPI) not reached the pontine surface, basilar artery stenosis was not observed on MRA. **(C)** LAOD. DWI showing PPI. MRA showing severe stenosis of the basilar artery.

We further classified pontine infarcts into three groups according to their etiological mechanism based on recently reported definitions and after evaluating the results of the diagnostic studies (4, 5): the patient was considered as large artery occlusive disease (LAOD) if basilar artery (BA) stenosis was present (>50%) assessed by means of MRA, CTA, or cerebral angiography. Basilar branch atheromatous disease (BAD) was considered if infarct lesions reached the surface of the pons (PPI) in the absence of vertebrobasilar large artery stenosis (>50%) and potential sources of cardioembolism. Small vessel disease (SVD) was assumed in patients with LPI in the absence of vertebrobasilar large artery stenosis (>50%) (Figure 1).

White matter hyperintensities (WMH) on FLAIR scans were not hypointense or were only faintly hypointense on T1-weighted images. Periventricular white matter hyperintensities (PVWMH) and deep white matter hyperintensities (DWMH) were scored on axial FLAIR images using the Fazekas scale (15). For this scale, the severity of WMH in different region is scored separately. Participants with severe WMH were defined as those with either PVWMH or DWMH rated as higher than 2 on the Fazekas scale. Analysis of MRI datasets was performed to determine WMH ratings by two well-trained neurologists (ZLX, NJ) who were blinded to the clinical data. Inter-rater reliability was assessed using a subset of 50 random study subjects. Kappa values for the inter-rater agreements were 0.85 for PVWMH, and 0.89 for DWMH.

Vascular imaging of the intracranial and extracranial arteries was required, and the identified images [magnetic resonance angiography (MRA), computed tomographic angiography (CTA), or cerebral angiography] were submitted to the SMART Coordinating Center. Stenosis of the intracranial and extracranial arteries on MRA, CTA, or angiogram was graded by the neuroradiologist (LML) using the NASCET criteria.

### Clinical characteristics and functional outcome

The following clinical information was reviewed from database of SMART study: age, gender, the history of hypertension, diabetes mellitus, hyperlipidemia, history of ischemic stroke and current smoking status. Hypertension was defined as SBP≥140 mm Hg or DBP ≥90 mm Hg, self-reported hypertension, or treatment with antihypertensive medication. Diabetes mellitus (DM) was defined as fasting serum glucose ≥7.0 mmol/l, self-reported diabetes, or use of oral blood sugar-lowering drugs or insulin; Hyperlipidemia was defined as total cholesterol >5.2 mmol/l or low-density lipoprotein-cholesterol >2.58 mmol/l, or self-reported hyperlipidemia. Current smoking status was defined as one who smoked at least one cigarette per day for more than 6 months before the stroke onset. To determine the neurological deficits and functional outcome, National Institutes of Health Stroke Scale (NIHSS) at admission and modified Rankin Scale (mRS) at 6-month follow-up were reviewed, the latter was further categorized as favorable when the patients remained independent (mRS 0-2) and poor when dependent (mRS 3-6).

### Statistical analysis

Statistical analyses were performed using the IBM SPSS statistics version 19 (SPSS Inc. Chicago, IL, USA). Statistical significance was set at a *P* < 0.05. Prevalence was compared among groups using Student's *t*-test or Kruskal–Wallis for continuous variables and a Chi-square test (or Fisher's exact test) for categorical data. The univariate and multivariate logistic regression model were used to compare the severity of WMH burden with different subtypes of AIPIs.

## Results

### Baseline data and vascular risk factors by AIPI etiology

Baseline data and vascular risk factors of the study population are displayed in the Table 1. Among a total of 175 participants, mean age was 63.44 ± 10.630 years, and 33% were female. Regarding the etiological classification, BAD was the most common subtype of AIPI (46.3 %, 81/175), followed by SVD (36.0%, 63/175) and LAOD (17.7%, 31/175). Female was significant more frequent in the BAD group as compared with the SVD group (*P* = 0.007), but no difference was found for age between two groups. The frequency of vascular risk factors was significantly different between groups. The BAD group had greater frequencies of hypertension, diabetes mellitus compared to the SVD group (*P* < 0.05). There was no significant difference in age, sex and vascular risk factors between BAD and LOAD group.

**Table 1:** Baseline data and risk factors by AIPI etiology.

	**SVD (*n* = 63) %**	**BAD (*n* = 81) %**	**LAOD (*n* = 31) %**	**BAD vs. SVD *P*-Value**	**BAD vs. LAOD *P*-Value**
Age, mean (SD)	62.22 (10.652)	64.56 (10.530)	63.00 (11.060)	0.178	0.441
Female, *n* (%)	13 (20.6%)	34 (42%)	10 (32.3%)	0.007	0.348
**RISK FACTORS**					
History of stroke, *n* (%)	18 (28.6%)	21 (25.9%)	9 (29.0%)	0.153	0.768
Smoking, *n* (%)	24 (38.1%)	27 (33.3%)	10 (32.3%)	0.555	0.914
Hypertension, *n* (%)	39 (61.9%)	65 (80.2%)	21 (67.7%)	0.01	0.128
Diabetes, *n* (%)	15 (23.8%)	33 (40.7%)	13 (41.9%)	0.025	0.883
Hyperlipidimia, *n* (%)	10 (15.9%)	17 (21%)	5 (17.2%)	0.347	0.505

### Clinical characteristics and functional outcome

The stroke severity (NIHSS score on admission) and outcome at 6 months are shown in Table 2. NIHSS score at admission were significantly higher in the BAD group as compared with the SVD group (*P* < 0.001), but no difference was found between BAD and LAOD group. A total of 36 cases were lost of follow-up at 6 months in this study, including 14 cases in BAD group, 10 cases in LAOD group and 12 cases in SVD group. Poor clinical outcome at 6 months was found in 13.7% (19/139) of patients and there was no difference among three groups.

**Table 2:** Stroke severity and functional outcome by AIPI etiology.

	**SVD**	**BAD**	**LAOD**	**BAD vs. SVD**	**BAD vs. LAOD**
				***P*-Value**	***P*-Value**
NIHSS on admission, mean (SD)	3.49 (3.115)	5.97 (3.924)	5.93 (4.690)	<0.001	0.616
mRS≥3 at 6 months *n* (%)	5 (9.8%)	13 (19.4%)	1 (4.8%)	0.153	0.112

### The association between WMH burden and etiology of AIPI

WMH severity was significant greater in SVD group compared to BAD group for the deep subcortical region; however, there was no difference for the periventricualr region. Multivariate logistic regression analyses adjusting for age and hypertension, was performed and showed that significant difference was still present for DWMH severity between the BAD and SVD groups (Table 3). There was no significant difference in either DWMH severity or PVWMH severity between the BAD and LAOD groups.

**Table 3:** DWMH and PVWMH severity by etiology of AIPI.

	**SVD (*n* = 63) , %**	**BAD (*n* = 81) , %**	**LAOD (*n* = 31) , %**	BAD vs. SVD *P*-Value^*^	BAD vs. LAOD *P*-Value^*^
Severe DWMH (Fazekas scale≥2)	12 (21.4%)	7 (9.3%)	5 (19.2%)	0.021	0.181
Severe PVWMH (Fazekas scale≥2)	24 (42.9%)	35 (46.7%)	9 (34.6%)	0.666	0.288

* *Adjusted for age and hypertension*.

## Discussion

The main finding of the present study was that WMH burden in BAD group was similar to the LAOD group, rather than to the SVD group, suggesting the underlying atherothrombotic nature of BAD. Few studies investigated the mechanisms of BAD, though Fisher and Caplan's first pathological reports in 1971 (2). Currently there is no consensus as to whether BAD should be included either among small vessel or among large vessel intracranial disease. Our results provided potential evidence that BAD represent a distinct etiological mechanism from SVD which may assist the secondary prevention decision in these patients.

Appropriate classification of ischemic stroke subtypes based upon causative mechanism is critical for guiding treatment decisions and determining the prognosis of individual patients. On the basis of pathological findings, the clinico-morphological correlation of isolated pontine infarcts has been investigated. According to the findings of the previous studies, paramedian infarcts that reached the basal surface of the ventral pons have been attributed to BAD, while small deep infarcts are thought to be due to lipohyalinotic SVD (4, 5). However, a standard etiological classification based on pontine infarct morphology has not been established. We validated an MRI-based etiological classification for pontine infarcts and to assess differences in vascular risk factors, radiological features, and outcomes from a national multi-center acute ischemic stroke study. Our results confirm the findings of previous studies that BAD was the most frequent etiology of AIPI followed by SVD and LAOD. Significant differences were found among three etiological subtypes with respect to baseline vascular risk factors and clinical characteristics. These results demonstrated the etiological classification system by pontine infarct morphology is reliable and can be easily used in clinical practice. Though significant difference was present in stroke severity at admission, etiological differences was not found to be relevant to long-term prognosis, which might be due to optimized secondary prevention according to the etiological subtypes in this cohort. Another reason might be a higher prevalence of missing data on follow-up, especially in LAOD group, which is one of the limitations of our study.

WMH has been accepted as a typical independent neuroimaging marker of SVD (16). The difference in WMH burden between SVD and BAD has rarely been previously studied and the results remain inconsistent. In the present study, less WMH burden were found in the BAD group compared to the SVD group and similar to LAOD, adjusting for age and hypertension. These results imply that atherosclerosis might be mainly involved in the pathophysiology of BAD. In agreement with our findings, a pervious study retrospectively reviewed the data from 805 small subcortical stroke patients, 114 of whom were attributed to BAD. Small vessel disease features such as leukoaraiosis and microbleeds were significantly less prevalent among BAD patients (17). In contrast, a subgroup study of SPS3 trial did not find any difference in WMH burden between participants with small deep and paramedian infarcts (18). Besides, WMH burden was further found to have a region difference between BAD and SAD. DWMH burden was significantly less in the BAD group as compared with the SVD group, but no difference was found for PVWMH burden between the same two groups. This is in line with the known mechanism of different region WMH. Different small vessel pathophysiology has been considered for WMH in different brain regions (19). Subcortical deep WMH is associated with lacunar infarction caused by small vessel lipohyalinosis, whereas periventricular WMH is associated with the complex small vessel abnormalities such as arteriosclerosis, venous collagenosis, and perivascular edema (20). The region-specific WMH burden difference between BAD and SVD again suggested that BAD represent a distinct etiological mechanism from SVD.

The present study has some limitations. First, the variability of the MRI sequences among the 47 hospitals randomized in the SMART study may have a potential impact on the delineation of ischemic lesions and WMH. Second, our study may be underpowered due to the limited number of events in this cohort with optimized secondary prevention and a higher prevalence of missing data on follow-up.

## Conclusions

Our results showed that BAD is the most frequent subtype of AIPI and reveal a relationship between region-specific WMH burden and etiological subtypes of pontine infarct which findings have provided insights into BAD pathophysiology. Management strategies for BAD should consider more intracranial atherosclerotic disease rather than small vessel disease. These findings might aware the clinicians the underlying different causes of pontine infarction and specific therapeutic approaches regarding the mechanism of pontine infarcts.

## Author contributions

LZ and JN contributed to the writing of the article. MY and YZ contributed to the collection of data. LZ and JN contributed to the rating of images. LC and BP contributed to the revising of the article.

### Conflict of interest statement

The authors declare that the research was conducted in the absence of any commercial or financial relationships that could be construed as a potential conflict of interest.

## References

[1] Bassetti C Bogousslavsky J Barth A Regli F. Isolated infarcts of the pons. Neurology (1996) 46:165–75. 10.1212/WNL.46.1.165 8559368

[2] Fisher CM Caplan LR. Basilar artery branch occlusion: a cause of pontine infarction. Neurology (1971) 21:900–5. 10.1212/WNL.21.9.900 5106254

[3] Caplan LR. Intracranial branch atheromatous disease: a neglected, understudied, and underused concept. Neurology (1989) 39:1246–50. 10.1212/WNL.39.9.1246 2671793

[4] Kumral E Bayulkem G Evyapan D. Clinical spectrum of pontine infarction. Clinical-MRI correlations. J Neurol. (2002) 249:1659–70. 10.1007/s00415-002-0879-x 12529787

[5] Erro ME Gállego J Herrera M Bermejo B. Isolated pontine infarcts: etiopathogenic mechanisms. Eur J Neurol. (2005) 12:984–8. 10.1111/j.1468-1331.2005.01119.x 16324092

[6] Kwon HM Kim JH Lim JS Park JH Lee SH Lee YS. Basilar artery dolichoectasia is associated with paramedian pontine infarction. Cerebrovasc Dis. (2009) 27:114–118. 10.1159/000177917 19039214

[7] Klein IF Lavallée PC Mazighi M Schouman-Claeys E Labreuche J Amarenco P. Basilar artery atherosclerotic plaques in paramedian and lacunar pontine infarctions: a high-resolution MRI study. Stroke (2010) 41:1405–9. 10.1161/STROKEAHA.110.583534 20538696

[8] Kim JS Yoon Y. Single subcortical infarction associated with parental arterial disease: important yet neglected sub-type of atherothrombotic stroke. Int J Stroke (2013) 8:197–203. 10.1111/j.1747-4949.2012.00816.x 22568537

[9] Petrone L Nannoni S Del Bene A Palumbo V Inzitari D. Branch atheromatous disease: a clinically meaningful, yet unproven concept. Cerebrovasc Dis. (2016) 41:87–95. 10.1159/000442577 26671513

[10] Bang OY. Intracranial atherosclerosis: current understanding and perspectives. J Stroke (2014) 16:27–35. 10.5853/jos.2014.16.1.27 24741562 PMC3961814

[11] Tamura A Yamamoto Y Nagakane Y Takezawa H Koizumi T Makita N. The relationship between neurological worsening and lesion patterns in patients with acute middle cerebral artery stenosis. Cerebrovasc Dis. (2013) 35:268–75. 10.1159/000348313 23548833

[12] Lin PC Chang FC Huang HC Tsai JY Lin YY Chung CP. Greater periventricular white matter hyperintensity severity in basilar artery branch atheromatous disease. BMC Neurol. (2017) 17:135. 10.1186/s12883-017-0918-y 28716089 PMC5514534

[13] Peng B Zhu Y Cui L Ni J Xu W Zhou L. Standard medical management in secondary prevention of ischemic stroke in China (SMART). Int J Stroke (2011) 6:461–5. 10.1111/j.1747-4949.2011.00648.x 21951412

[14] Peng B Ni J Anderson CS Zhu Y Wang Y Pu C. Implementation of a structured guideline-based program for the secondary prevention of ischemic stroke in China. Stroke (2014) 45:515–9. 10.1161/STROKEAHA.113.001424 24385269

[15] Fazekas F Chawluk JB Alavi A Hurtig HI Zimmerman RA. MR signal abnormalities at 1.5 T in Alzheimer's dementia and normal aging. Am J Roentgenol. (1987) 149:351–6. 10.2214/ajr.149.2.351 3496763

[16] Wardlaw JM Smith C Dichgans M. Mechanisms of sporadic cerebral small vessel disease: insights from neuroimaging. Lancet Neurol. (2013) 12:483–97. 10.1016/S1474-4422(13)70060-7 23602162 PMC3836247

[17] Nah HW Kang DW Kwon SU Kim JS. Diversity of single small subcortical infarctions according to infarct location and parent artery disease: analysis of indicators for small vessel disease and atherosclerosis. Stroke (2010) 41:2822–7. 10.1161/STROKEAHA.110.599464 20966406

[18] Wilson LK Pearce LA Arauz A Anderson DC Tapia J Bazan C. Morphological classification of penetrating artery pontine infarcts and association with risk factors and prognosis: the SPS3 trial. Int J Stroke (2016) 11:412–9. 10.1177/1747493016637366 26956031

[19] Kim KW MacFall JR Payne ME. Classification of white matter lesions on magnetic resonance imaging in elderly persons. Biol Psychiatry (2008) 64:273–80. 10.1016/j.biopsych.2008.03.024 18471801 PMC2593803

[20] Mäntylä R Aronen HJ Salonen O Pohjasvaara T Korpelainen M Peltonen T. Magnetic resonance imaging white matter hyperintensities and mechanism of ischemic stroke. Stroke (1999) 30:2053–8. 10.1161/01.STR.30.10.2053 10512906

